# ATP indirectly stimulates hippocampal CA1 and CA3 pyramidal neurons *via* the activation of neighboring P2X7 receptor-bearing astrocytes and NG2 glial cells, respectively

**DOI:** 10.3389/fphar.2022.944541

**Published:** 2022-07-22

**Authors:** Ying Zhang, Hai-Yan Yin, Patrizia Rubini, Peter Illes, Yong Tang

**Affiliations:** ^1^ School of Acupuncture and Tuina, Chengdu University of Traditional Chinese Medicine, Chengdu, China; ^2^ International Collaborative Center on Big Science Plan for Purinergic Signaling, Chengdu University of Traditional Chinese Medicine, Chengdu, China; ^3^ Rudolf Boehm Institute for Pharmacology and Toxicology, University of Leipzig, Leipzig, Germany; ^4^ Key Laboratory of Sichuan Province for Acupuncture and Chronobiology, Chengdu University of Traditional Chinese Medicine, Chengdu, China

**Keywords:** astrocytes, oligodendrocytes, microglia, pyramidal neurons, hippocampus, mouse

## Abstract

There is ongoing dispute on the question whether CNS neurons possess ATP-sensitive P2X7 receptors (Rs) or whether only non-neuronal cells bear this receptor-type and indirectly signal to the neighboring neurons. We genetically deleted P2X7Rs specifically in astrocytes, oligodendrocytes and microglia, and then recorded current responses in neurons to the prototypic agonist of this receptor, dibenzoyl-ATP (Bz-ATP). These experiments were made in brain slice preparations taken from the indicated variants of the P2X7R KO animals. In hippocampal CA3, but not CA1 pyramidal neurons, the deletion of oligodendrocytic (NG2 glial) P2X7Rs abolished the Bz-ATP-induced current responses. In contrast to the Bz-ATP-induced currents in CA3 pyramidal neurons, current amplitudes evoked by the ionotropic glutamate/GABA_A_R agonists AMPA/muscimol were not inhibited at all. Whereas in the CA3 area NG2 glia appeared to mediate the P2X7R-mediated stimulation of pyramidal neurons, in the CA1 area, astrocytic P2X7Rs had a somewhat similar effect. This was shown by recording the frequencies and amplitudes of spontaneous excitatory currents (sPSCs) in brain slice preparations. Bz-ATP increased the sPSC frequency in CA1, but not CA3 pyramidal neurons without altering the amplitude, indicating a P2X7R-mediated increase of the neuronal input. Micro-injection of the selective astrocytic toxin L-α-aminoadipate into both hippocampi, or the *in vitro* application of the GABA_A_R antagonistic gabazine, completely blocked the frequency increases of sPSCs. Hence, CA1 and CA3 pyramidal neurons of the mouse did not possess P2X7Rs, but were indirectly modulated by astrocytic and oligodendrocytic P2X7Rs, respectively.

## Introduction

P2X7 receptors (Rs) constitute a subtype of the ligand-gated P2XR superfamily and are activated by high concentrations of extracellular ATP released from the intracellular space in response to various injurious or inflammatory conditions (Surprenant et al., 1996; North, 2002). They are able to transit from the non-selective cationic-channel mode to a large membrane pore which allows the diffusion of molecules up to 900 Da [e.g., ATP, glutamate, GABA (Sperlágh et al., 2006), and fluorescent dyes (Bartlett et al., 2014)], according to their concentration gradient. While originally P2X7R-channels were thought to dilate on long-lasting or repetitive contact with ATP (Virginio et al., 1999), or alternatively to recruit the membrane channel pannexin-1 as a conduit (Pelegrin and Surprenant, 2006), more recently it was found that P2X7Rs permit the passage of large cationic molecules immediately from their initial activation, but at a much slower pace than that of the small cations Na^+^, K^+^, and Ca^2+^ (Harkat et al., 2017; Di Virgilio et al., 2018).

P2X7Rs are located at peripheral (lymphocytes, macrophages, dendritic cells) and central (microglia) immunocytes, where they initiate the synthesis and subsequent outflow of inflammatory mediators, such as cytokines, chemokines, proteases, reactive oxygen/nitrogen species, and the excitotoxic ATP and glutamate (Kigerl et al., 2009; Kettenmann et al., 2011; Zhao et al., 2022). In the CNS, P2X7Rs occur at the highest density at microglia, but there is also unequivocal evidence for their existence at astrocytes and oligodendrocytes (Zhao et al., 2021). With respect to their localization at neurons there has been a long-lasting controversy on the issue whether P2X7Rs directly or indirectly (*via* the release of astrocytic, oligodendrocytic or microglial signaling molecules) cause neuronal effects at the cellular and systemic levels (Illes et al., 2017; Miras-Portugal et al., 2017).

Molecular biology methods also failed to dissolve these controversies. On the one hand it was published that in different brain regions of a P2X7R conditional humanized mice, the respective mRNA was specifically expressed in glutamatergic pyramidal neurons of the CA3 hippocampal area (Metzger et al., 2017). In addition, P2X7Rs were identified in major non-neuronal lineages throughout the brain (i.e., astrocytes, oligodendrocytes, microglia). On the other hand, the generation of a P2X7 EGFP-tagged transgenic mouse and investigations on the expression of the respective protein in the brain, failed to indicate a neuronal localization of this receptor in the hippocampal CA1/CA3 pyramidal neurons, and dentate gyrus granule cells (Kaczmarek-Hajek et al., 2018). However, P2X7R-EGFP co-stained with microglial, oligodendrocytic and astrocytic immunohistochemistry markers, whereas there was no co-staining with any of the standard neuronal markers. A handicap of these studies was that in both cases the functional identification of the P2X7R-mRNA or -protein was missing.

It has to be mentioned already at this stage, that besides astrocytes and oligodendrocytes, NG2 glial cells emerged during the last decades as a new type of neuroglial cells in the CNS (Seifert and Steinhäuser, 2018; Bedner et al., 2020; Zhao et al., 2021). They express in addition to the classic antigenic markers (e.g., oligodendrocyte transcription factor; Olig2) also the proteoglycan NG2. These cells are also referred to as oligodendrocyte precursor cells (OPCs) because in the white, but not grey matter they differentiate into myelinating oligodendrocytes (Nishiyama et al., 2016). In the hippocampus the number of NG2 cells amounts to about 20%–25% of that of astrocytes (Degen et al., 2012).

The aim of our present study was to fill this gap, by recording dibenzoyl ATP (Bz-ATP) currents from hippocampal CA1/CA3 neurons in brain slices of mice whose P2X7Rs were genetically deleted in astrocytes, oligodendrocytes (NG2 glia) or microglia. In addition, we searched for assumedly presynaptic P2X7Rs at the terminals of the Schaffer collaterals and mossy fibers ending at CA1 and CA3 pyramidal cells, respectively. There was no functional evidence for the existence of P2X7Rs at the investigated neuronal structures; by contrast, these receptors were associated with astrocytes or oligodendrocytes releasing signaling molecules onto the targeted pyramidal neurons upon the activation of their P2X7Rs.

## Materials and methods

### Animals

In most experiments, humanized P2X7R knockout (KO) mice (P2rX7^tm1,2Jde^; MGI:6203042), generated on a mixed 129S2/Sv×C57BL/6J background (Charles-River, Wilmington, MA, United States; Metzger et al., 2017) as well as their wild-type (WT) controls were used. The mice were kindly donated by Jan M. Deussing, Max Planck Institute of Psychiatry, Munich, Germany. In addition, C57BL/6J mice were used for the measurement of spontaneous postsynaptic current (sPSC) amplitude and frequency. Both strains of mice were housed under standard laboratory conditions and maintained on a 12 h light-dark cycle with food and water provided *ad libitum*. The animal study was reviewed and approved by the Institutional Review Board of the Chengdu University of Traditional Chinese Medicine, Chengdu, China (protocol code, DC2472, 1 April 2019).

To generate astrocyte-specific deletion of P2X7Rs in mice (P2rX7flox/flox; GFAP-Cre/Esr1 mice), we crossed mice bearing P2rX7 floxed alleles to GFAP-Cre/Esr1 mice to obtain P2rX7flox/+; GFAP-Cre/Esr1 mice. In the next generation, we used P2rX7flox/+, GFAP-Cre/Esr1 mice to breed with P2rX7 flox/flox mice to generate P2rX7flox/flox; GFAP-Cre/Esr1 mice. The subsequent experiments were performed by using progenies of P2rX7 flox/flox mice crossed with P2rX7flox/flox; GFAP-Cre/Esr1 mice. A similar procedure was used to generate mice whose P2X7Rs were specifically deleted in their oligodendrocytes/NG2 glia (P2rX7flox/flox; Olig2-Cre/Esr1) or microglia (P2rX7flox/flox; CX3CR1-Cre/Esr1). We used P2rX7flox/flox mice as controls. The three types of Cre-mice were generous gifts from Yong-Jun Chen (Guangzhou University of Traditional Chinese Medicine, Guangzhou, China). It has to be noted that the yield of astrocyte-, oligodendrocyte-, and microglia-specific KO mice was quite low; they amounted only to 15%–30% of the final number of littermates.

### Patch-clamp recordings in hippocampal slices and selection of neurons/astrocytes for experimentation

Coronal hippocampal slices were prepared from the brain of 10–15 days old mice and were used for patch-clamp recordings as described previously (Ficker et al., 2014; Khan et al., 2019). After decapitation, the brain was placed into ice-cold and oxygenated (95% O_2_+5% CO_2_) artificial cerebrospinal fluid (aCSF) of the following composition (in mM): KCl 2.5, NaCl 126, MgCl_2_ 1.3, CaCl_2_ 2.4, NaH_2_PO_4_ 1.2, NaHCO_3_ 25 and glucose monohydrate 11. Hippocampal slices of mice were cut at the thickness of 200 μm by using a vibratome (VT1200S; Leica Biosystem, Muttenz, Switzerland). To create low divalent cationic conditions (low X^2+^), MgCl_2_ was omitted from the medium and the CaCl_2_ concentration was decreased to 0.5 mM. The brain slices were superfused in an organ bath with 95% O_2_+5% CO_2_-saturated low X^2+^ aCSF at 37°C for 30 min, and then superfusion was continued at room temperature (20–24°C) throughout.

Neurons and astrocytes in the CA1 and CA3 region were visualized by using a 40× water immersion objective (Axio Examiner.A1, Carl Zeiss, Oberkochen, Germany). Patch pipettes were filled with intracellular solution of the following composition (in mM): K-gluconic acid 140, NaCl 10, MgCl_2_ 1, HEPES 10, EGTA 11, Mg-ATP 1.5, Li-GTP 0.3; pH 7.3 adjusted with KOH. Pipettes (4–9 MΩ for neurons; 9–12 MΩ for astrocytes) were pulled by a horizontal micropipette puller (P-1000; Sutter Instruments, Novato, CA, United States) from borosilicate capillaries.

Whole-cell current-clamp and voltage-clamp recordings were made by a patch-clamp amplifier (MultiClamp 700B; Molecular Devices, San Jose, CA, United States). In the current-clamp mode of recording, astrocytes were discriminated from neurons by their failure to fire action potentials in response to depolarizing current injection (−80 to −760 pA, in 60 pA increments). Most non-spiking cells belonged to the passive class (typical astrocytes) and only very few to the variably rectifying class; there were no in- or outwardly rectifying cells at all (Oliveira et al., 2011; Zhao et al., 2022). This is a clear distinction from microglia and oligodendrocytes, which both express under resting conditions an inwardly rectifying current pattern (Boucsein et al., 2000; Pérez-Samartín et al., 2017). Furthermore, earlier experiments showed that when Lucifer yellow diffused from the recording pipette into electrophysiologically-identified astrocytes, these cells also stained immunohistochemically for the astrocytic marker S100β (Oliveira et al., 2011).

### Recordings of agonist-induced currents and spontaneous postsynaptic current frequency/amplitude

In the voltage-clamp recording mode of the amplifier, the holding potential of astrocytes was set to −80 mV and that of neurons to −70 mV. Agonists were applied locally by means of a computer-controlled solenoid valve-driven pressurized superfusion system (VC38; ALA Scientific Instruments, Farmingdale, NY, United States). The drug application tip touched the surface of the brain slice and was placed 100–150 µm from the patched cell. Agonists were diluted and applied in low X^2+^ solution for 10 s every 3 min, and each agonist was administered twice; the mean current response was calculated for statistical evaluation. pClamp 10.4 software (Molecular Devices) was used to store the recorded data, to perform online analysis/filtering, and to trigger the application system used.

Spontaneous postsynaptic currents (sPSCs) composing of both action potential-induced and spontaneous vesicular glutamate/GABA release were recorded from neurons at the holding potential of −70 mV. They were analyzed by means of the pClamp 10.4 software package, by detecting amplitudes exceeding the detection threshold set at three times the standard deviation above the baseline noise of the recordings. sPSCs were recorded for 5 min in the absence and for another 5 min in the presence of Bz-ATP. Then, the reversibility of the Bz-ATP effect was demonstrated by recording sPSCs for another 5 min after washing out Bz-ATP. Bz-ATP was investigated on preparations taken from L-α-aminoadipate-treated mice (see below) or on preparations superfused with aCSF containing the GABA_A_R antagonist gabazine (10 µM) throughout. Superfusion with gabazine started at least 5 min before beginning the experiment.

### *In vivo* drug-treatment protocols to selectively inactivate astrocytes or microglia

In order to selectively abrogate astrocytes, the astrocytic toxin L-α-aminoadipate (20 μg/μl stock, 2 μl at a constant rate of 0.4 μl/min) was infused into the bilateral hippocampus (Lima et al., 2014). The animals were anesthetized with isoflurane (5% induction, 2% maintenance) and fixed on a stereotactic platform (RWD Life Science, San Diego, CA, United States). The stereotactic coordinates for the positioning of the guide cannula into the bilateral hippocampus were −2 mm anterior-posterior, ± 1.5 mm lateral to bregma, and 1.5 mm below the surface of the skull. A micro-syringe (5 μl; Hamilton Company, Reno, NV, United States) was used for injection, and constant rate infusion was delivered by a stereotactic injection syringe pump (RWD Life Science). At the end of the infusion, the dummy cannula was retained at the injection place to allow sufficient diffusion. To selectively inactivate microglia, mice were injected intraperitoneally (i.p.) with minocycline (40 mg/kg; Bassett et al., 2021). Animals were decapitated and their hippocampal slices were prepared the 3rd day after either pre-treatment.

### Drugs and chemicals

The following drugs were used: L-α-aminoadipic acid, 2′ (3′)-O-(4-benzoylbenzoyl)adenosine-5′-triphosphate tri (triethylammonium) salt (Bz-ATP), gabazine, minocycline hydrochloride, muscimol, *N*-methyl-D-aspartic acid (NMDA) (*S*)-α-amino-3-hydroxy-5-methylisoxazole-4-propionic acid (S-AMPA) (Sigma-Aldrich, St. Louis, MO, United States). Chemicals for solutions were also from Sigma-Aldrich.

### Statistical analysis

All data were expressed as means ± SEM of *n* observations, where *n* stands for the number of cells recorded in hippocampal slices taken from at least four animals. There was no *a priori* sample size calculation, but instead we have set the number of measurements to 10 in all experimental groups. GraphPad Prism9 was used for the construction of Figures and for statistical evaluations. We tested for and found that, when using parametric tests, all sampled distributions satisfied the normality and equal variance criteria. Multiple comparisons between data were performed in case of their normal distribution by either one-way or two-way ANOVA, as appropriate, followed by the Tukey’s or Dunnett’s multiple comparison’s tests, as appropriate. Two data sets were compared using the parametric Student’s *t*-test or the non-parametric Mann-Whitney rank sum test, as appropriate. A probability level of 0.05 or less was considered to be statistically significant.

## Results

### Effects of general and cell-specific deletion of P2X7Rs on responses to the Bz-ATP-induced stimulation of hippocampal astrocytes and neurons

**FIGURE 1:**
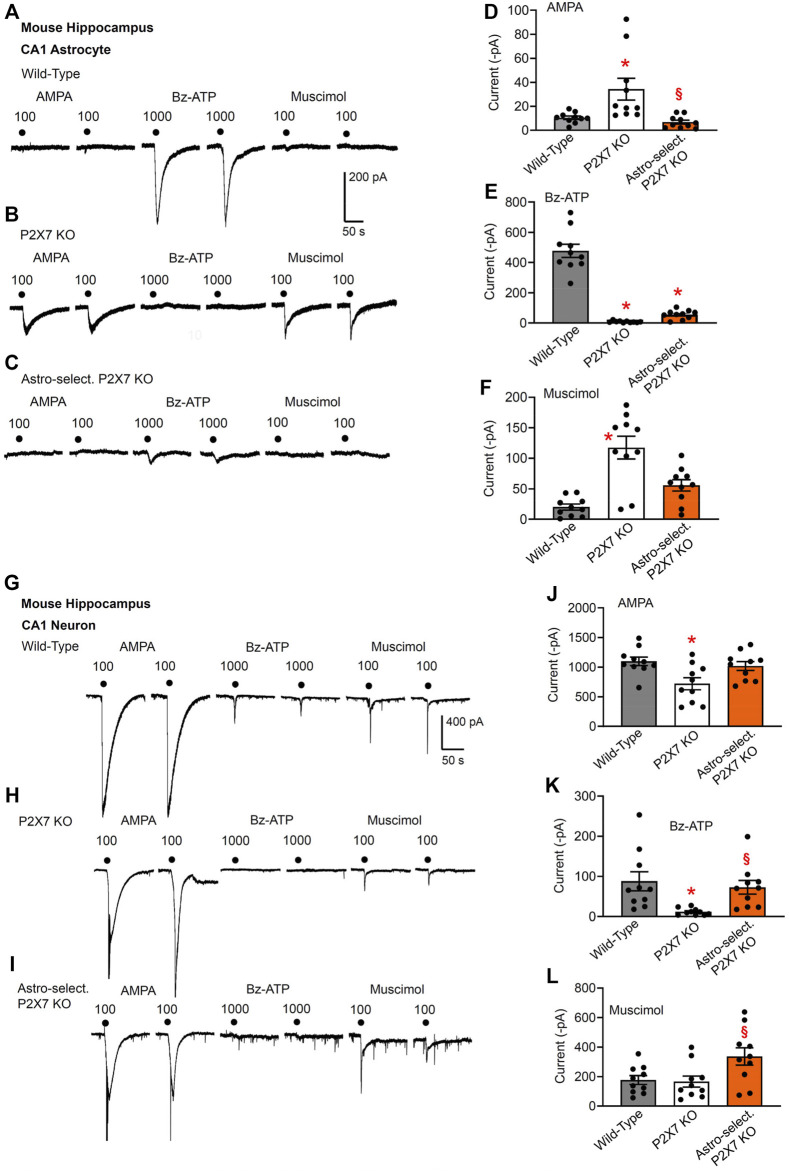
Current responses to ionotropic receptor agonists in the CA1 area of the mouse hippocampus. Hippocampal brain slices of 10–15 day-old wild-type (WT) mice, as well as mice whose P2X7Rs were genetically deleted either in all cells, or selectively in astrocytes. AMPA (100 µM), Bz-ATP (1,000 µM) and muscimol (100 µM) were locally applied every 3 min, each agonist twice. **(A–F)** The membrane currents were recorded in astrocytes by means of the whole-cell patch-clamp method at a holding potential of −80 mV. The superfusion medium was in this and all further experiments a low X^2+^-containing aCSF solution (see Methods). Representative tracings in **(A–C)**. Effects of AMPA **(D)**, Bz-ATP **(E)**, and muscimol **(F)** in the three types of animals. Each column indicates the mean ± SEM of current amplitudes of 10 cells from at least four mice both in this Fig. as well as in all subsequent Figs. of the study. * *p* < 0.05; statistically significant difference from results in wild-type animals (AMPA, *F*_2,18_ = 8.875, *p* = 0.0092; Bz-ATP; *F*_2,18_ = 92.93, *p* < 0.0001; muscimol, *F*_2,18_ = 19.74, *p* < 0.0001; two-way ANOVA followed by the Tukey’s test). ^§^ *p* < 0.05; statistically significant difference from general P2X7R KO animals (AMPA, *p* = 0.0030; Bz-ATP, *p* < 0.0001; muscimol, *p* = 0.0026; two-way ANOVA followed by the Tukey’s test). **(G–L)** Similar experiments as in **(A–F)**, but instead of astrocytes, pyramidal neurons were used for the investigations. The holding potential was −70 mV. Representative tracings in **(G-I)**. Effects of AMPA **(J)**, Bz-ATP **(K)**, and muscimol **(L)** in the three types of animals. Mean ± SEM current amplitudes. * *p* < 0.05; statistically significant difference from results in wild-type mice (AMPA, F_2,18_ = 5.253, *p* = 0.0170; Bz-ATP, F_2,18_ = 6.852, *p* = 0.0069; two-way ANOVA followed by the Tukey’s test). ^§^ *p* < 0.05; statistically significant difference from general P2X7R KO animals (AMPA, *p* = 0.0170; Bz-ATP, *p* = 0.0303; muscimol, *F*_2,18_ = 3.801, *p* = 0.0420; two-way ANOVA followed by the Tukey’s test).

In a first series of experiments, we recorded by means of the whole-cell patch-clamp technique agonist-induced membrane currents of hippocampal CA1 astrocytes in brain slices (Figures 1A–F). We applied selective agonists for the AMPA-type excitatory glutamate receptor (AMPA, 100 µM), the inhibitory amino acid receptor GABA_A_ (muscimol, 100 µM) and the non-selective agonist for excitatory P2X7Rs (Bz-ATP, 1,000 µM).

**FIGURE 2:**
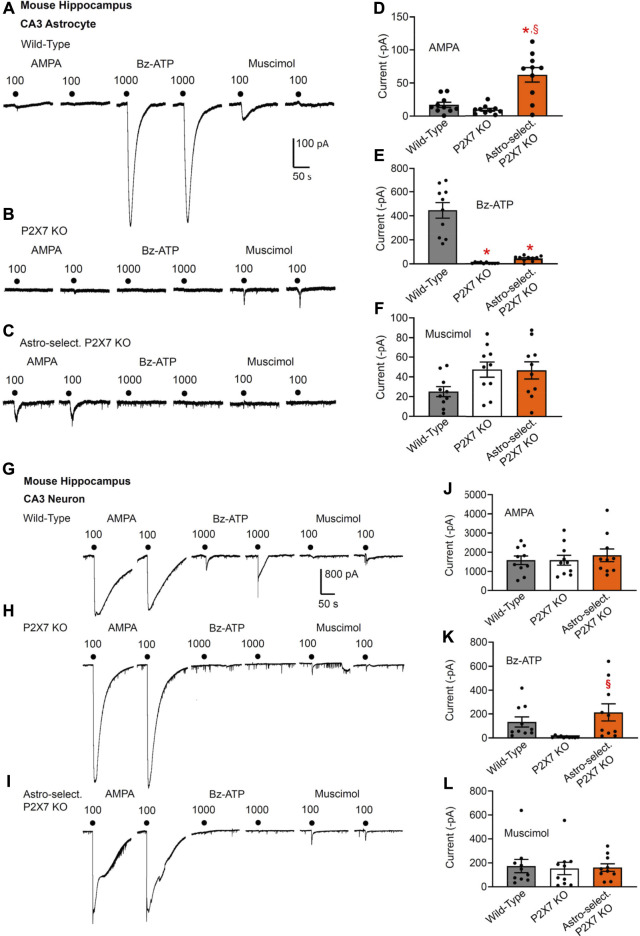
Current responses to ionotropic receptor agonists in the CA3 area of the mouse hippocampus. Hippocampal brain slices of 10–15 day-old wild-type mice, as well as mice whose P2X7Rs were genetically deleted either in all cells, or selectively in astrocytes. AMPA (100 µM), Bz-ATP (1,000 µM) and muscimol (100 µM) were locally applied every 3 min, each agonist twice. **(A–F)** The membrane currents were recorded in astrocytes by means of the whole-cell patch-clamp method at a holding potential of −80 mV. Representative tracings in **(A–C)**. Effects of AMPA **(D)**, Bz-ATP **(E)**, and muscimol **(F)** in the three types of animals. Mean ± SEM current amplitudes. * *p* < 0.05; statistically significant difference from results in wild-type animals (AMPA, *F*_2,18_ = 21.61, *p* = 0.0002; Bz-ATP, *F*_2,18_ = 42.03, *p* < 0.0001; two-way ANOVA followed by the Tukey’s test). ^§^ *p* < 0.05; statistically significant difference from general P2X7R KO animals (AMPA, *p* = 0.0030; Bz-ATP, *p* < 0.0001; two-way ANOVA followed by the Tukey’s test). **(G–L)** Similar experiments as in **(A–F)**, but instead of astrocytes, pyramidal neurons were used for the investigations. The holding potential was −70 mV. Representative tracings in **(G-I)**. Effects of AMPA **(J)**, Bz-ATP **(K)**, and muscimol **(L)** in the three types of animals. Mean ± SEM current amplitudes. ^§^ *p* < 0.05; statistically significant difference from general P2X7R KO animals (Bz-ATP, *F*_2,18_ = 3.748, *p* = 0.0360; two-way ANOVA followed by the Tukey’s test).

**FIGURE 3:**
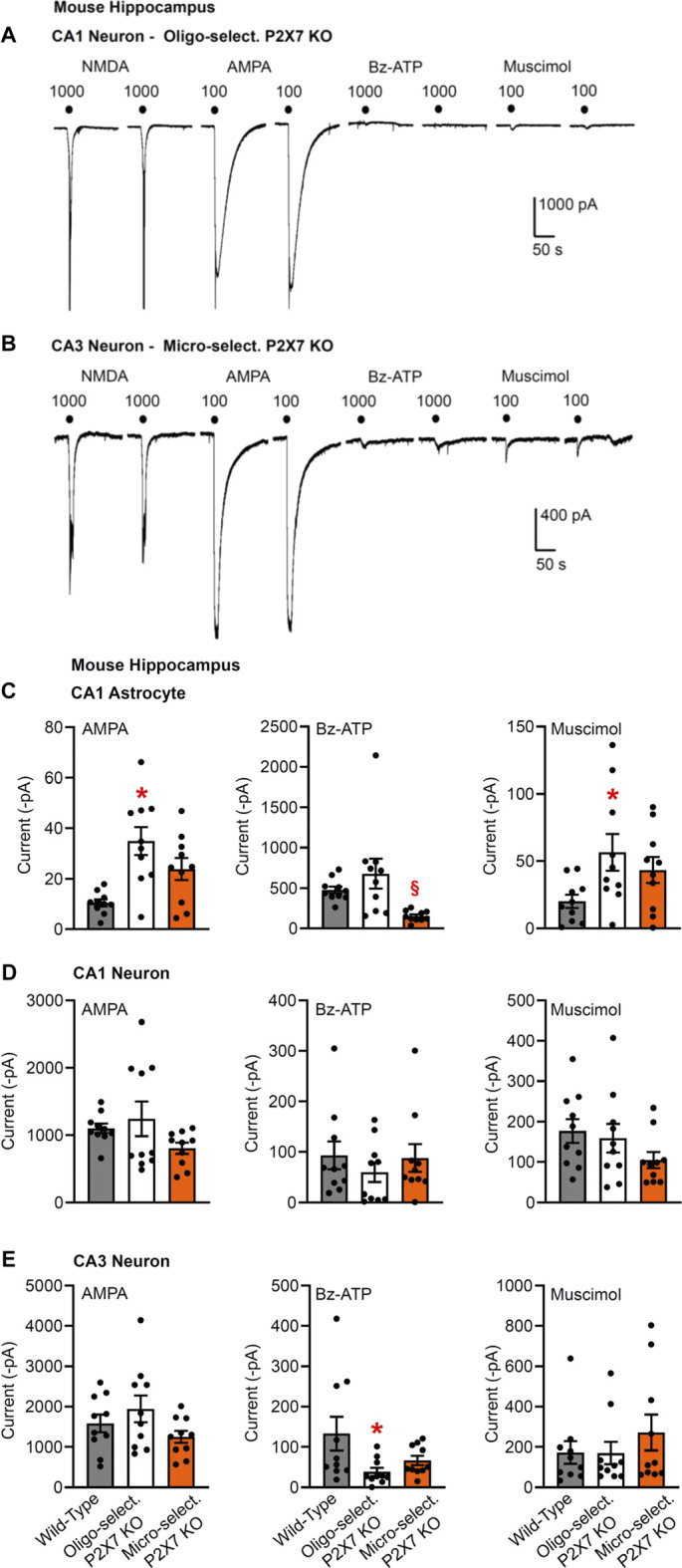
Current responses to ionotropic receptor agonists in the CA1 and CA3 areas of the mouse hippocampus. Hippocampal brain slices of 10–15 day-old wild-type mice, as well as mice whose P2X7Rs were genetically deleted in all cells, or selectively in oligodendrocytes or microglia. NMDA (100 µM), AMPA (100 µM), Bz-ATP (1,000 µM) and muscimol (100 µM) were locally superfused every 3 min, each agonist twice. The membrane currents were recorded in astrocytes by means of the whole-cell patch-clamp method at a holding potential of −80 mV, and in neurons at a holding potential of −70 mV. Representative tracings in neurons **(A,B)**. **(C–E)** Mean ± SEM current amplitudes. **(C)** Effects of AMPA, Bz-ATP, and muscimol in CA1 astrocytes of the three types of animals. * *p* < 0.05; statistically significant difference from results in wild-type animals (AMPA, *F*_2,18_ = 7.565, *p* = 0.0030; muscimol, *F*_2,18_ = 3.553, *p* < 0.0427; two-way ANOVA followed by the Tukey’s test). **(D,E)** Similar experiments as in C, but instead of astrocytes, CA1 or CA3 pyramidal neurons were used for the investigations. Mean ± SEM current amplitudes. * *p* < 0.05; statistically significant difference from results in wild-type mice (Bz-ATP, *F*_2,18_ = 3.632, *p* = 0.0432; two-way ANOVA followed by the Tukey’s test.

**FIGURE 4:**
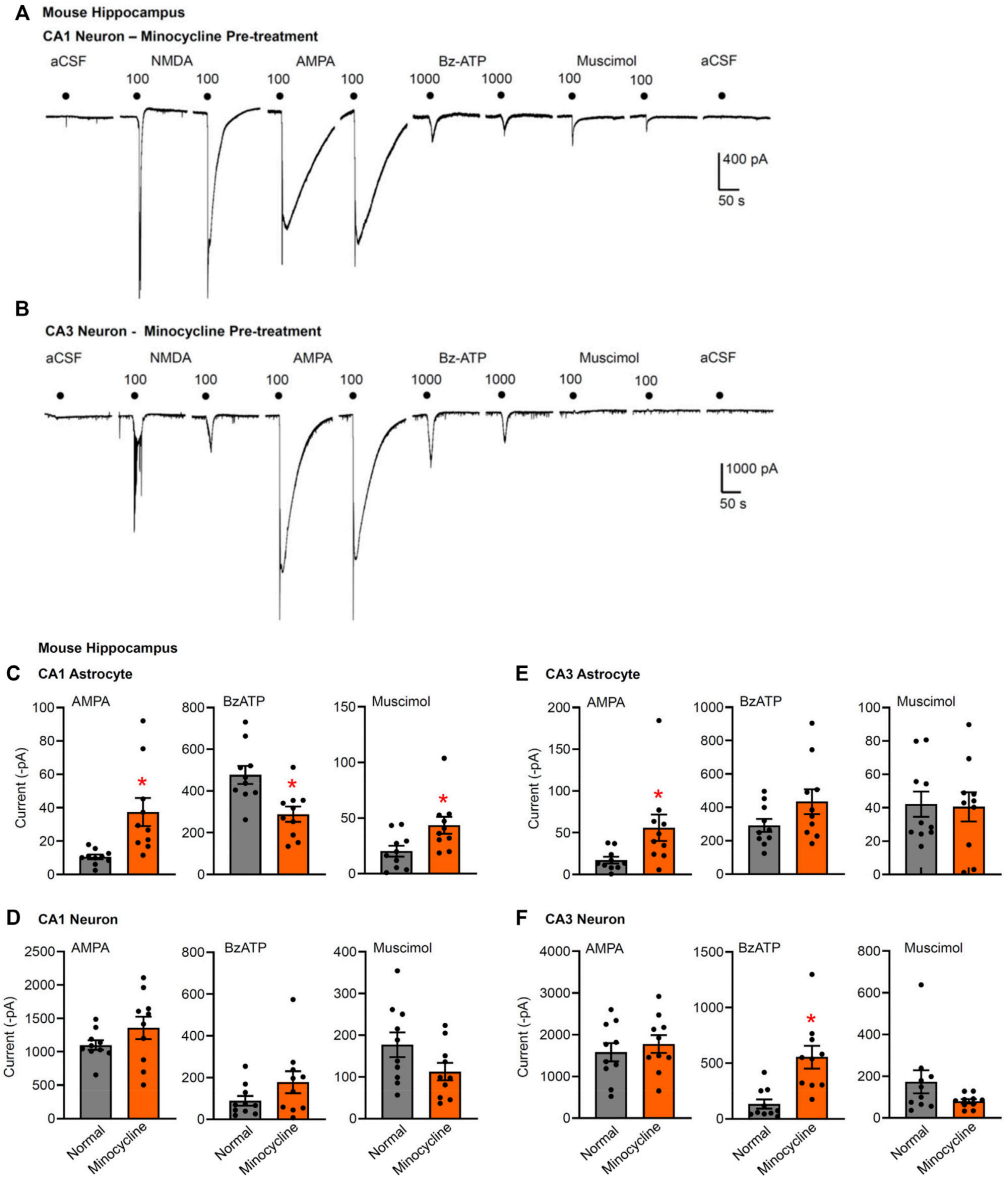
Current responses to ionotropic receptor agonists in the CA1 and CA3 areas of the mouse hippocampus. Hippocampal brain slices of 10–15 day-old wild-type mice without or with pre-treatment with minocycline (40 mg/kg, i.p. 3 days before the preparation of the slices). NMDA (100 µM), AMPA (100 µM), Bz-ATP (1,000 µM), muscimol (100 µM), and an equivalent amount of solvent (low X^2+^-containing aCSF) were locally superfused every 3 min, each agonist twice. The membrane currents were recorded in astrocytes by means of the whole-cell patch-clamp method at a holding potential of −80 mV, and in neurons at a holding potential of −70 mV. Representative tracings in neurons **(A,B)**. **(C–F)** Mean ± SEM current amplitudes. **(C)** Effects of AMPA, Bz-ATP, and muscimol in CA1 astrocytes of the three types of animals. * *p* < 0.05; statistically significant difference from results in wild-type animals (AMPA, *p* < 0.0001, Mann-Whitney test; Bz-ATP, *p* = 0.0036, unpaired *t*-test; muscimol, *p* = 0.0205, unpaired *t*-test). **(D)** Effects of AMPA, Bz-ATP, and muscimol in CA1 neurons of the three types of animals. **(E)** Effects of AMPA, Bz-ATP, and muscimol in CA3 astrocytes of the three types of animals. * *p* < 0.05; statistically significant difference from results in wild-type mice (AMPA, *p* = 0.0279, unpaired *t*-test). **(F)** Effects of AMPA, Bz-ATP, and muscimol in CA3 neurons of the three types of animals * *p* < 0.05; statistically significant difference from results in wild-type mice (Bz-ATP, *p* = 0.0013, unpaired *t*-test).

In this type of experiments, the same concentrations of the agonists were used throughout (Figures 1–4). AMPA, Bz-ATP and muscimol were applied twice in succession, and the mean of the two current responses was used for statistical evaluation. In order to potentiate the effect of Bz-ATP in WT astrocytes, a low X^2+^-containing superfusion medium was used, which is known to largely increase P2X7R currents (Jiang, 2009). It is also noteworthy that Bz-ATP activates P2X1/P2X3Rs with an even higher efficiency than its supposed target site, the P2X7R; however, Bz-ATP is 10–30 times more potent at P2X7Rs than ATP itself and is considered therefore to be a prototypic agonist for this latter receptor-type (Anderson and Nedergaard, 2006; Illes et al., 2021). In spite of using Bz-ATP and a low cation-containing superfusion medium, a high agonistic concentration had to be applied, a finding, which agrees with the well-known low sensitivity of P2X7Rs to ATP when compared with that of the residual subtypes of the P2XR superfamily (Surprenant et al., 1996; Sperlágh et al., 2006). A further reason for the need to use high agonist concentrations was that mouse, in comparison with rat P2X7Rs, only weakly respond to ATP/Bz-ATP, due to differences in the amino acid composition of the ectodomains of the two receptor orthologs (Young et al., 2007). In fact, when concentration-response curves for Bz-ATP were constructed on astrocytes in brain slices of the pre-frontal cortex (Oliveira et al., 2011) or hippocampus (Zhao et al., 2022), a much lower agonist-sensitivity was found in the mouse than in the rat preparation. Actually, Bz-ATP at 1,000 µM elicited reproducible, submaximal current responses well suitable for further work.

Notwithstanding the necessity to utilize Bz-ATP concentrations in the millimolar range, a selective activation of P2X7Rs occurred, because in CA1 astrocytes of the general P2X7R KO mouse, no current response to this agonist was observed at all (compare Figures 1A,E with Figures 1B,E). In addition to the general deletion of P2X7Rs in all cells of the mouse, also astrocyte-selective P2X7R KO mice were generated; in this case, the very small Bz-ATP current response confirmed the success of this manipulation (Figures 1C,E). The residual response to Bz-ATP may be due to the activation of a non-P2X7 receptor-type. The absence of P2X7Rs was accompanied by a compensatory increase in AMPA- and muscimol-sensitivities in the general knockouts (Figures 1B,D,F). For such an increase in the mentioned receptor-sensitivities apparently a complete absence of P2X7Rs is needed; a partial absence of this latter receptor-type is not sufficient.

In spite of the poor effect of AMPA and muscimol in WT astrocytes, both muscimol and especially AMPA caused large currents in CA1 neurons (Figures 1G,J,L). According to our expectations, the general deletion of P2X7Rs abolished the Bz-ATP (1,000 µM)-induced current in CA1 neurons (Figures 1H,K). Similarly, the astrocyte-selective deletion of this receptor failed to cause any change of the Bz-ATP-induced current amplitudes, excluding the possibility that an astrocytic signaling molecule mediates neuronal activation (Figures 1I,K). There was no dramatic reduction of the responses to AMPA or muscimol either in the general knockouts or in the astrocyte-specific ones although the AMPA-induced current responses did decrease in the general knockouts (Figures 1G–J).

As discussed above, in CA1 astrocytes, the effects of AMPA and muscimol were increased in the general P2X7 KO mice relative to the responses to these agonists in WT and astrocyte-specific KO preparations; no similar changes occurred in neurons of the CA1 area. We have to confess that we do not know why the astrocytic and neuronal effects differed from each other. It is quite possible that there was no immediate and corresponding modulation of the excitatory and inhibitory amino acid-induced neuronal effects, but the astrocytic alterations would cause long-term changes due to a modified homeostatic function of this cell type (e.g., Illes et al., 2019; Verkhratsky et al., 2021).

Results in CA3 astrocytes and neurons roughly confirmed the data obtained in the analogous cells of the CA1 area (Figures 2A–L). The astrocyte-selective deletion of P2X7Rs once again failed to alter the effect of Bz-ATP in neurons, whereas this manipulation strongly inhibited the effect of Bz-ATP in astrocytes (compare Figure 2E with Figure 2K). However, the original recording in Figure 2I shows one of the neurons which after the astrocyte-selective genetic deletion of P2X7Rs did not respond to Bz-ATP at all (see Figure 2K). The larger current responses to AMPA in astrocytes of astrocyte-selective KO animals in comparison with their WT counterparts can be again explained with some compensatory mechanism, in that AMPA takes over the function of P2X7Rs under these conditions (Figure 2A).

Then, we checked the change of Bz-ATP-sensitivity in CA1/CA3 neurons after oligodendrocyte (NG2 glia)-selective deletion of P2X7Rs (Figures 3A,C–E). We did not find a statistically significant change in the CA1 pyramidal cell-sensitivity (although in the half of the total number of investigated cells there was no response to Bz-ATP at all Figures 3A,D); while in the analogous cells of the CA3 area the effect of Bz-ATP was almost abolished. This finding clearly indicates that in contrast to astrocytes, CA3 oligodendrocyte-like NG2 glia appear to secrete a signaling molecule onto the neighboring neurons upon stimulation of their P2X7Rs.

We also confirmed that the oligodendrocyte-selective deletion of P2X7Rs has no effect on the Bz-ATP-induced currents of CA1 astrocytes (Figure 3C), in contrast to the strong blockade of these responses in the CA1 astrocytes after the astrocyte-selective deletion of P2X7Rs (Figures 1C,E). In agreement with the compensatory increase of AMPA- and muscimol-induced currents in astrocytes of general P2X7R KO mice (Figures 1B,D,F), similar changes occurred also after the selective deletion of oligodendrocytic P2X7Rs (Figure 3C).

Eventually, a microglia-selective deletion of P2X7Rs did not alter AMPA-, Bz-ATP, or muscimol-evoked current responses in CA1/CA3 neurons of the hippocampus (Figures 3B,D,E). The original tracings in Figures 3A,B, show in addition that another ionotropic glutamate receptor agonist, NMDA also causes prominent effects in these neurons in a low X^2+^-containing superfusion medium. This is possibly in concord with the well-known blockade of NMDA receptors by extracellular Mg^2+^ and their apparent disinhibition in the nominal absence of external Mg^2+^.

It is interesting to note that the Bz-ATP-evoked currents in CA1 astrocytes appeared to decrease after a microglia-selective deletion of P2X7Rs (Figure 3C), although, as already mentioned, the neuronal current responses to Bz-ATP were not changed at all. A similar decrease of the CA1 astrocytic currents was also observed after treatment with minocycline, known to block microglial activation (see below).

### Effects of interference by minocycline with microglial activation on Bz-ATP-induced currents in hippocampal astrocytes and neurons

Minocycline is commonly used to inhibit microglial activation (Bassett et al., 2021). However, microglia exert dual functions, that is pro-inflammatory and anti-inflammatory ones (Illes et al., 2020). In addition, to the classically activated M1 microglial phenotype, releasing the previously mentioned pro-inflammatory mediators and causing neurodegeneration, the alternatively activated M2-phenotype clears cellular debris through phagocytosis and releases numerous protective factors [IL-4, IL-13, nerve growth factor (NGF), fibroblast growth factor (FGF)], thereby contributing to neuroregeneration (Kettenmann et al., 2013). It was reported that minocycline selectively inhibits the microglial polarization to a pro-inflammatory state, and leaves the pro-regenerative functions undisturbed (Kobayashi et al., 2013).

In the light of these results we investigated the modulation by i.p. minocycline of the astrocytic and neuronal effects of Bz-ATP in the hippocampal CA1 and CA3 area of the mouse. A somewhat unexpected finding of these series of experiments was that Bz-ATP-induced current responses in CA3 neurons increased, rather than decreased in minocycline-treated brain slices (Figures 4B,F). In addition, the same responses in CA1 responses also showed some tendency to increase although this change was not statistically significant (Figures 4A,D). In both cell-types, we also tested the application of the solvent aCSF to confirm that the mechanical strain of the superfusion did not cause any current response (Figures 4A,B). The rises of the AMPA and muscimol currents in the CA1 astrocytes, assumedly compensating the reduced P2X7R activity, could be again observed (Figures 1, 4C,E).

Our explanation for the potentiation of the Bz-ATP responses in the CA3 neurons is based on the selective blockade of the pro-inflammatory M1 phenotype of microglia, under the assumption that certain cytokines and growth factors amplify the outflow of ATP from its astrocytic pools or alternatively promote the sensitivity of various P2Rs to the ATP released (Heine et al., 2006; Heine et al., 2007; Oliveira et al., 2016). In consequence, an unexpected facilitation of P2X7R functions may develop on the CA3 pyramidal neurons.

### Increase by Bz-ATP of the frequency but not amplitude of spontaneous postsynaptic currents in CA1 hippocampal neurons

**FIGURE 5:**
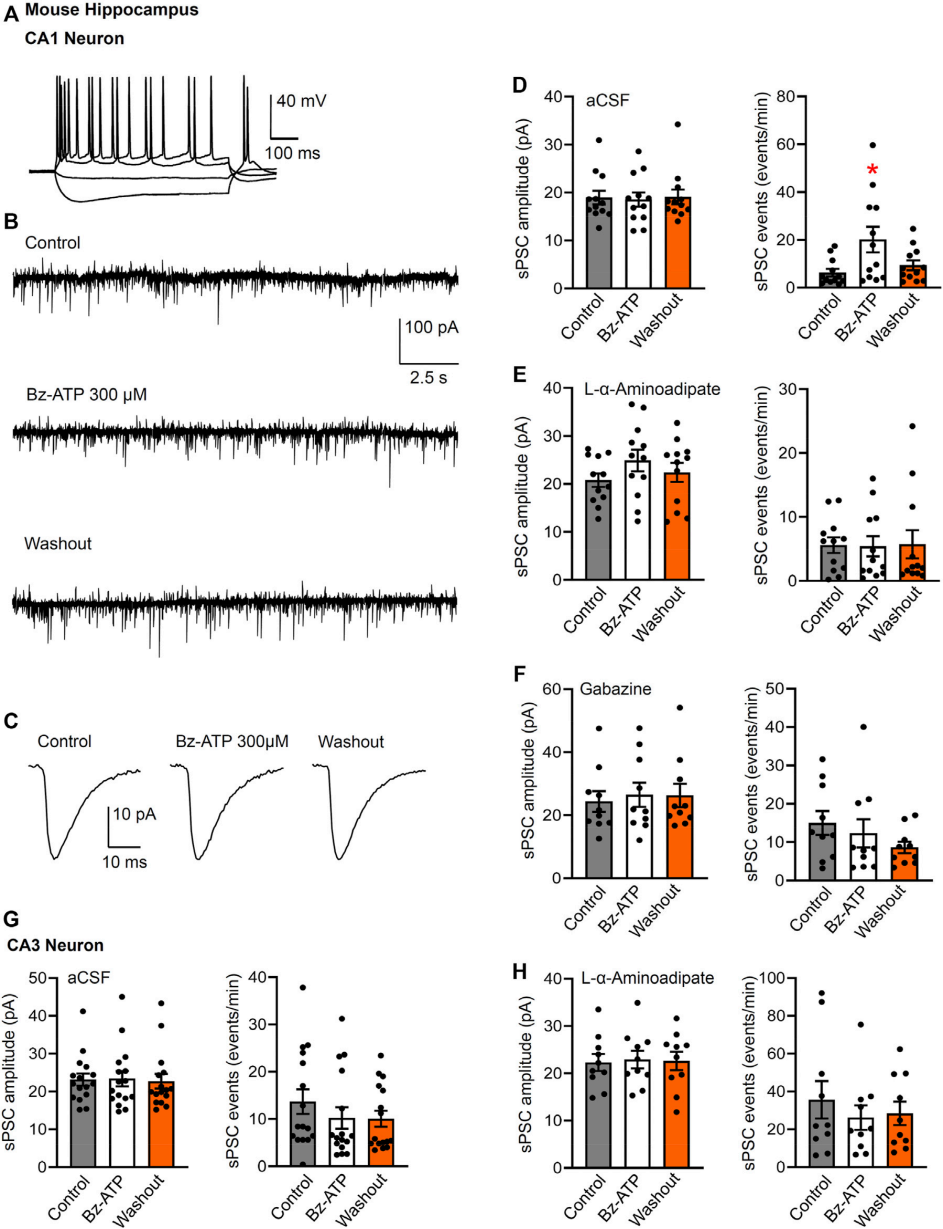
Spontaneous postsynaptic currents (sPSCs) in CA1 and CA3 neurons in brain slices of mice; effects of Bz-ATP after or without L-α-aminoadipate pre-treatment. **(A)** Identification of CA1 neurons by current injection into whole-cell patch-clamped cells. **(B–D)** Effect of Bz-ATP (300 µM) on the amplitude and frequency of sPSCs in a low X^2+^-containing aCSF. Representative recordings of sPSCs before, during and after superfusion with Bz-ATP for 5 min **(B)**. Representative, averaged sPSC amplitudes before, during and after a 5-min superfusion with Bz-ATP **(C)**. **(D)** Mean ± SEM sPSC amplitudes and frequencies in brain slices prepared from untreated mice. * *p* < 0.05; statistically significant difference from control sPSCs (*F*_2,33_ = 4.477, p = 0.0142; one-way ANOVA followed by the Dunnett’s test). **(E)** Mean ± SEM sPSC amplitudes and frequencies in brain slices prepared from mice pre-treated with L-α-aminoadipate (intrahippocampal application, 20 μg; see Methods). **(F)** Mean ± SEM sPSC amplitudes and frequencies in brain slices prepared from untreated mice continuously superfused with aCSF containing gabazine (10 µM). Pre-treatment schedule with the selective astrocytic toxin L-α-aminoadipate, or blockade of GABA_A_Rs by gabazine, both abolished the effect of Bz-ATP. Experiments were made also in CA3 neurons of hippocampal brain slices prepared from untreated **(G)** and L-α-aminoadipate pre-treated **(H)** mice.

After having dealt with the possibly postsynaptic neuronal P2X7Rs, we turned our attention to their assumedly presynaptic counterparts. In fact, we discriminated by the usual technique CA1 pyramidal neurons from neighboring astrocytes by injecting depolarizing current pulses into the cells and deciding that action potential firing is a neuronal property (Figure 5A). Then, we have set the holding potential to −70 mV and recorded sPSCs before, during, and after the application of Bz-ATP (300 µM) for 5 min each (Figure 5B). Bz-ATP increased the mean sPSC frequency, without altering the mean sPSC amplitude (Figures 5C,D). This effect may be considered to be an indication for the boosting of the spontaneous release of glutamate/GABA from the glutamatergic mossy fiber nerve terminals and GABAergic interneurons (Khan et al., 2019). However, a third possibility also exists, namely that the source of these transmitters are P2X7R possessing astrocytes sending their processes to the CA1 pyramidal neurons.

This latter assumption was supported by the abolishment of the Bz-ATP effect in brain slices of mice previously treated by intrahippocampal injection of the astrocytic toxin L-α-aminoadipate (Figure 5E). L-α-aminoadipate is a glutamate homologue which selectively ablates astrocytes without influencing neuronal density and function (Khurgel et al., 1996; Gochenauer and Robinson, 2001). In addition, the GABA_A_R antagonist gabazine (10 µM) also inhibited the effect of Bz-ATP on the frequency of sPSCs (Figure 5F). In contrast to the observed effects of Bz-ATP on the sPSC frequency of CA1 pyramidal cells, there was no comparable influence on CA3 pyramidal cells and in consequence L-α-aminoadipate pre-treatment did not cause a further effect in the latter area of the brain (Figures 5G,H). In consequence, it is suggested that Bz-ATP increases the exocytotic release of astrocytic signaling molecules rather than that of their neuronal counterparts (see Discussion).

## Discussion

The main finding of this study is that the oligodendrocyte-selective genetic deletion of P2X7Rs in the hippocampal CA3 area resulted in an indirect reduction of the P2X7R-mediated current responses at the neighboring pyramidal neurons. Hence, these data exclude the assumption that P2X7R-mRNA in CA3 pyramidal neurons of mice indicates the presence of the respective receptor protein (Metzger et al., 2017). The validity of our findings is strengthens by the fact that we used the mouse line generated by Metzger et al. (2017), in which the humanized P2rX7 allele being accessible to spatially and temporally controlled Cre recombinase-mediated inactivation allowed a cell-type specific deletion of P2X7Rs. Thus, we caution against drawing conclusions from the presence of mRNA for any type of functional protein in a given cell-type.

In previously available and broadly used P2rX7^−/−^ mouse models the knockout strategy did not result in complete inactivation of the P2rX7 gene (Kaczmarek-Hájek et al., 2012; Bartlett et al., 2014). In the mouse line established by Glaxo, the P2rX7 gene was disrupted by targeted insertion of a lacZ/Neo reporter cassette into exon 1, which manipulation, however, left the translation of the highly functional P2rX7k splice variant unaltered (Nicke et al., 2009). In the mouse line produced by Pfizer, a portion of exon 13 has been deleted and replaced by a neomycin resistance cassette (Solle et al., 2001). In this case several C-terminally truncated variants escaped deletion (Masin et al., 2012). In contrast to these conventional KO mice, none of the described P2rX7 splice variants evaded the null allele in the humanized P2rX7 KO mouse of Metzger et al. (2017).

However, our data are only in partial accordance with another study concluding that P2X7Rs are probably absent in any neuron of the CNS (Kaczmarek-Hajek et al., 2018). As mentioned earlier, this study reported the generation of a P2rX7 BAC transgenic mice which allowed the visualization of EGFP-tagged P2X7Rs in the brain. The authors criticized previous results obtained with a transgenic reporter mouse [Tg (p2rX7 EGFP)FY174Gsat] which showed a wide neuronal P2X7R expression in the brain (Engel et al., 2012; Jimenez-Pacheco et al., 2013). Kaczmarek-Hajek et al. (2018) believe that alterations in gene structure introduced into the GenSat P2rX7 BAC EGFP mouse influenced post-transcriptional and translational regulatory mechanisms. In fact, they ascribed all neuronal effects to indirect stimulation mediated by oligodendrocytic signaling molecules. Our data perfectly agree with this suggestion, but only in case of the CA3 hippocampal region; in CA1 pyramidal neurons, astrocytes, rather than oligodendrocytes released GABA in response to stimulation of their P2X7Rs.

A whole plethora of findings strongly support the notion that astrocytes are able to secrete signaling molecules and thereby are capable of regulating neuronal circuit functions and animal behavior (Illes et al., 2019; Hirrlinger and Nimmerjahn, 2022). The tripartite synapse hypothesis suggests that presynaptic neuronal elements, postsynaptic dendritic specializations and astrocytic processes that contact or even enwrap the synapse, together form a mutually interacting unit (Araque et al., 1999; Pascual et al., 2005). Neurotransmitters originating from presynaptic axon terminals may induce the release of gliotransmitters (glutamate, GABA, ATP, D-serine, taurine), which act at the postsynaptic specializations of neurons. The release mechanisms of ATP from astrocytes is through Ca^2+^-dependent exocytosis (Lalo et al., 2014; Pankratov and Lalo, 2014), most likely from lysosomes (Zhang et al., 2007). However, a non-exocytotic release by means of channel molecules, including connexin hemichannels, pannexin-1 channels, maxi-anion channels, volume-regulated ion channels, Bestrophin1 channels, and the calcium homeostasis modulator 1 (CALHM1) is also possible (Dahl, 2015; Illes et al., 2020).

Specifically, in CA1 pyramidal cell-containing brain slices, Bz-ATP caused inward currents both in neurons and in neighboring astrocytes (Ficker et al., 2014). The effect of Bz-ATP was depressed by the selective P2X7R antagonist A-438079, and neuronal, but not astrocytic Bz-ATP currents were strongly inhibited by a combination of the ionotropic glutamate receptor antagonists AP-5 and CNQX, as well as the GABA_A_R antagonist gabazine. This finding has borne out the conclusion that P2X7Rs are possibly situated at astrocytes which release glutamate/GABA to stimulate pyramidal neurons. In fact, astrocytic P2X7Rs have been shown to mediate the release glutamate (Duan et al., 2003; Fellin et al., 2006), GABA (Wirkner et al., 2005), and ATP itself (Suadicani et al., 2006; Illes et al., 2017).

Bz-ATP potentiated the frequency of sPSCs (consisting of glutamatergic, GABAergic and ATPergic synaptic potentials) in CA1 pyramidal cells in a hippocampal slice preparation of rats, without altering the amplitude of these spontaneous events (Khan et al., 2019). The selective P2X7R antagonist A-438079 abolished the potentiation by Bz-ATP, assigning this effect to the simulation of the respective receptors. However, the question still remained to be answered, whether the P2X7Rs are localized at glutamatergic or GABAergic neurons or alternatively at neighboring astrocytes. The GABA_A_R antagonist gabazine and the selective astrocytic toxin fluorocitrate (Clarke, 1991), both abolished the effect of Bz-ATP when applied under *in vitro* conditions. Further, a series of electrophysiological experiments excluded the participation of GABAergic interneurons in the stratum radiatum as possible sources of external GABA and left the astrocytic release as an undisputable opportunity (Khan et al., 2019). Hence, it was concluded that the release of GABA is potentiated by P2X7R activation and astrocytes rather than GABAergic interneurons are involved in this effect.

Our present experiments fully agree with this conclusion, by confirming the blockade of a previously observed Bz-ATP-induced increase of sPSC frequency both by locally applied gabazine and by pre-treatment of mice with another selective astrocytic toxin, L-α-aminoadipate (Khurgel et al., 1996; Gochenauer and Robinson, 2001). Hence, CA1 pyramidal cells of both rats and mice appear to be innervated by Schaffer collaterals subjected to GABAergic modulation by gliotransmitter release from local astrocytic processes.

Oligodendrocytes just as astrocytes, also possess P2X7Rs (Matute, 2008; Zhao et al., 2021). P2X7Rs in oligodendrocytes are highly permeable to Ca^2+^ and prolonged activation of these receptors is lethal to differentiated oligodendrocytes in culture and to mature oligodendrocytes in isolated optic nerves *in vitro* and *in vivo*. *In vitro* ischemia achieved by replacing O_2_ by N_2_, external glucose by sucrose, and by adding iodoacetate to the incubation medium to block glycolysis, induced inward currents in cultured oligodendrocytes, which could be reversed by the P2X7R antagonist Brilliant Blue, the ATP degrading enzyme apyrase, and by blockers of pannexin hemichannels (Domercq et al., 2010). Because oligodendrocytes constitute the myelin sheath of central fiber tracts, they are localized in the white matter insulating individual axons in order to permit spatially separated and rapid conduction of actions potentials.

In the hippocampus, oligodendrocyte precursor cells (OPCs) occur, giving rise to mature oligodendrocytes in the white matter, but functioning as a separate type of glial cells, the so called NG2 glia, in grey matter (Seifert and Steinhäuser, 2018; Bedner et al., 2020; see also Introduction). Bz-ATP was reported to increase intracellular Ca^2+^ in cultured NG2 glia in a manner fully dependent on extracellular Ca^2+^ and blocked by the P2X7R antagonistic oxidized ATP (Agresti et al., 2005a; Agresti et al., 2005b). Our findings implicate that in the CA3 area of the hippocampus P2X7R-bearing NG2 glia mediate the purportedly neuronal effects of Bz-ATP.

As a fourth type of non-neuronal cells capable of releasing a whole range of signaling molecules in response to P2X7R stimulation, microglia have to be taken into consideration (Kigerl et al., 2009; Kettenmann et al., 2011). Microglia are secreting ATP both by exocytosis (Imura et al., 2013) and by the shedding of extracellular vesicles (Graner, 2018). In addition, microglia were reported to release glutamate within a range of further neurotoxic molecules (Illes et al., 2020; Lindhout et al., 2021). While especially the upregulation of A2ARs occupied by adenosine, an enzymatic degradation product of ATP, drives the transition of resting/surveilling microglia to its amoeboid phenotype, P2X7Rs are involved in the secretory functions of these phagocytic microglial cells. Many studies have focused on the interactions between the cellular processes of surveilling microglia and synaptic elements, including axonal boutons and dendritic spines (Wu et al., 2015; Weinhard et al., 2018). Recently, it was reported that microglia senses ATP *via* P2Y12Rs, thereafter a microglia-dependent production of adenosine ensues, and adenosine suppresses neuronal responses *via* its A2AR-type (Badimon et al., 2020). However, under the present conditions we did not find evidence for the involvement of microglial P2X7Rs in neuronal responses to Bz-ATP.

In conclusion, we supply evidence for the participation of astrocytic and oligodendrocytic P2X7Rs in the indirect stimulation of certain neuronal structures in the hippocampus of mice, but cannot exclude the presence of P2X7Rs at the residual CNS neurons. We have to point out as well that our conclusions are based on the finding that 1,000 µM of Bz-ATP causes near-maximum current amplitudes in pyramidal neurons of the mouse hippocampus (Zhao et al., 2022) (also reported for organotypic spinal cord slices of the mouse substantia gelatinosa; Gao et al., 2017), and a further increase of the Bz-ATP concentration would not unmask a previously absent P2X7R-sensitivity.

Another caveat is due to the age of the animals. It was reported that in the mouse neocortex, stimulation of neuronal afferents triggers complex glial synaptic currents (GSCs mediated by NMDA, P2X and AMPA-Rs as well as glutamate transporters) (Lalo et al., 2011; Boué-Grabot and Pankratov, 2017). The P2X component of GSCs is the smallest in young, maximal in adult, and once more decreases in old mice. Somewhat similar, the exocytotic release of ATP from astrocytes exhibits an age-dependent decrease (Lalo et al., 2014). In view of these complexities it has to be concluded, that our findings relate to P10-15 mice and any extrapolation to older mice is only hypothetical, especially because ATP is considered to be an ontogenetically and phylogenetically primitive signaling molecule (Gao et al., 2017; Verkhratsky, 2021).

## Data Availability

The original contributions presented in the study are included in the article, further inquiries can be directed to the corresponding authors.
